# Preoperative Radiomics Analysis of 1p/19q Status in WHO Grade II Gliomas

**DOI:** 10.3389/fonc.2021.616740

**Published:** 2021-07-06

**Authors:** Ziwen Fan, Zhiyan Sun, Shengyu Fang, Yiming Li, Xing Liu, Yucha Liang, Yukun Liu, Chunyao Zhou, Qiang Zhu, Hong Zhang, Tianshi Li, Shaowu Li, Tao Jiang, Yinyan Wang, Lei Wang

**Affiliations:** ^1^ Department of Neurosurgery, Beijing Tiantan Hospital, Capital Medical University, Beijing, China; ^2^ Beijing Neurosurgical Institute, Capital Medical University, Beijing, China; ^3^ Department of Pathology, Beijing Tiantan Hospital, Capital Medical University, Beijing, China; ^4^ Department of Neuroradiology, Beijing Tiantan Hospital, Capital Medical University, Beijing, China

**Keywords:** radiomics, 1p/19q co-deletion, low grade glioma, nested cross-validation, machine learning

## Abstract

**Purpose:**

The present study aimed to preoperatively predict the status of 1p/19q based on radiomics analysis in patients with World Health Organization (WHO) grade II gliomas.

**Methods:**

This retrospective study enrolled 157 patients with WHO grade II gliomas (76 patients with astrocytomas with mutant IDH, 16 patients with astrocytomas with wild-type IDH, and 65 patients with oligodendrogliomas with mutant IDH and 1p/19q codeletion). Radiomic features were extracted from magnetic resonance images, including T1-weighted, T2-weighted, and contrast T1-weighted images. Elastic net and support vector machines with radial basis function kernel were applied in nested 10-fold cross-validation loops to predict the 1p/19q status. Receiver operating characteristic analysis and precision-recall analysis were used to evaluate the model performance. Student’s *t*-tests were then used to compare the posterior probabilities of 1p/19q co-deletion prediction in the group with different 1p/19q status.

**Results:**

Six valuable radiomic features, along with age, were selected with the nested 10-fold cross-validation loops. Five features showed significant difference in patients with different 1p/19q status. The area under curve and accuracy of the predictive model were 0.8079 (95% confidence interval, 0.733–0.8755) and 0.758 (0.6879–0.8217), respectively, and the F1-score of the precision-recall curve achieved 0.6667 (0.5201–0.7705). The posterior probabilities in the 1p/19q co-deletion group were significantly different from the non-deletion group.

**Conclusion:**

Combined radiomics analysis and machine learning showed potential clinical utility in the preoperative prediction of 1p/19q status, which can aid in making customized neurosurgery plans and glioma management strategies before postoperative pathology.

## Introduction

Molecular pathology is valuable for determining strategies for treating gliomas and for predicting the prognostic outcome (1). Patients without chromosome 1p/19q co-deletions showed poor overall and progression-free survival (2, 3). Neurosurgeons intended to protect the fundamental functions for patients whose eloquent cortices or white matter were invaded by gliomas, especially gliomas with 1p/19q co-deletions (4, 5). Although the association between prognosis and extent of tumor resection in gliomas with 1p/19q co-deletion remains controversial, some studies have indicated that gross total resection showed better prognosis than that in subtotal resection (6, 7). Nevertheless, other studies have shown no significant difference (8, 9). Undoubtedly, the prediction of the 1p/19q status before performing neurosurgery can aid in making customized neurosurgery plans and glioma management.

Radiomics is a novel practice for detecting the intrinsic imaging features of tumors (10–12). By using radiomics analysis (1), which converts sparse magnetic resonance imaging (MRI) data into big data, we can acquire a large amount of imaging information that is otherwise invisible to the naked eye in multiple dimensions (13–15). Moreover, machine learning is a prevalent artificial intelligent measurement to make classifications. The status of some well-known biomarkers has been accurately predicted in glioma patients, such as *IDH* mutations (16), *ATRX* mutations (17), p53 status (18), and the expression index of Ki-67 (19). However, an accurate and effective method for the preoperative prediction of 1p/19q co-deletion is lacking.

Consequently, in the current study, we retrospectively enrolled patients with low-grade glioma [grade II in pathological criteria of the World Health Organization (WHO, 2016)] (1). By using radiomics analysis, we acquired relevant neuroimaging features and then built a predictive model for 1p/19q status through a machine learning method.

## Materials and Methods

### Patients

**Figure 1:**
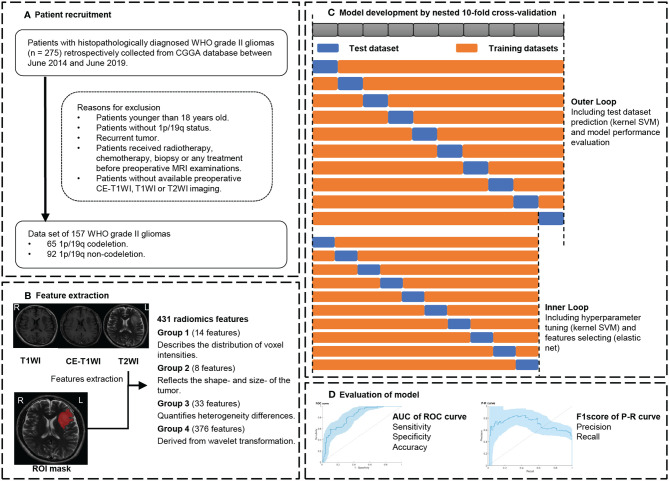
Workflow. **(A)** Patient recruitment strategy. **(B)** 431 features were extracted from region of interest (ROI) on each magnetic resonance imaging (MRI) sequence. **(C)** To compute a 10 × 10-fold nested cross-validation scheme, data were split into 9 training sets and a test set in the outer loop. The inner loop included hyperparameter tuning and features selection in the training datasets. After feature selection, the model with optimal parameters was used for prediction in the test set. This procedure developed 10 different models with specific sets of features and hyperparameters. **(D)** receiver operating characteristic (ROC) analysis and precision and recall (P-R) analysis were used for model performance evaluation. CGGA, Chinese Glioma Genome Atlas database; CE-T1WI, contrast-enhanced T1-weighted imaging; T2WI, T2-weighted imaging; AUC, area under the curve.

In this retrospective study, we collected the clinical data and biological information regarding the gliomas from the Chinese Glioma Genome Atlas (CGGA, http://www.cgga.org.cn/) database (from June 2014 to June 2019; **Figure 1**). A total of 157 patients formed a consecutive series following the selection criteria: (a) older than 18 years; (b) histopathological diagnosis of primary World Health Organization (WHO) II gliomas; (c) no preoperative treatment or biopsy; and (d) available preoperative contrast-enhancement T1-weighted images (CE-T1WI), T1WI, and T2-weighted images (T2WI). The tumor subtypes of WHO grade II gliomas were identified according to the WHO 2016 classification (20). The information of IDH mutations was acquired from the CGGA database, and the details of the measurements can be found in the **Supplementary Materials**.

### Imaging Acquisition and Tumor Segmentation

MRI scans were performed using a Trio 3.0-T scanner (Siemens, Erlangen, Germany) to obtain the MR images and typically included axial T1WI (TE, 15 ms; TR, 450 ms; slice thickness, 5 mm), T2WI (TE, 110 ms; TR 5800 ms; slice thickness, 5 mm), and CE scans using 0.1 mM/kg gadopentetate dimeglumine (Ga-DTPA injection, Beilu Pharma, Beijing, China) (TE, 15 ms; TR, 450 ms; slice thickness, 5 mm), with field of view 240 × 188 mm^2^. The tumor masks were manually segmented on T2WI by two experienced neurosurgeons (ZF and SF >5 years of experience in diagnosis) using MRIcro software (http://www.mccauslandcenter.sc.edu/mricro/), and a third senior neuroradiologist (SL, >20 years of experience) reevaluated the tumor masks and made the final decision when discrepancies were >5%.

### Fluorescence In Situ Hybridization of 1p/19q Co-Deletion

The 4-μm formalin-fixed paraffin-embedded tissue sections, which were obtained from neurosurgical operations, were deparaffinized, permeabilized, and hybridized. Dual-color fluorescence was performed using Vysis (Illinois, USA) of 1p36/1q25 and 19q13/19p13 according to the standardized procedure (21) and evaluated in at least 200 non-overlapping nuclei with intact morphology. We defined >25% of nuclei showing DNA loss as having chromosome loss. Co-deletion of 1p/19q was defined as loss of both 1p and 19q in tumor cells; 1p/19q non-codeletion included tumors with maintenance in 1p or 19q.

### Extraction of Radiomic Signatures

All the sequences of the MRIs and ROIs for each patient were resliced to high-resolution (1.0-mm isotropic) images using MATLAB, and the T1WI and CE-T1WI were then registered to the T2WI using the SPM8 software (http://www.fil.ion.ucl.ac.uk/spm/software/spm8). The z-score transformations were used to normalize the brain MRI signal intensity values into standardized intensity ranges. These procedures helped avoid bias from heterogeneity and sequences. Thereafter, the radiomic features were extracted from the tumor masks based on different types of MRI sequences using an automated approach in MATLAB (details shown in **Supplementary Material**) (22). A total of 431 radiomic features were included for each sequence. The feature groups included 14 first-order statistics (pertaining to the distribution of signal intensity of images, Group 1), eight shape- and size-based features (Group 2), 33 textural features (pertaining to intratumoral heterogeneity, Group 3), and 376 wavelet features that were derived from group 1 and group 3 features *via* wavelet decomposition (using directional low-pass and high-pass filtering; the original features were decomposed into eight decompositions, group 4).

### Feature Selection Method: Elastic Net

Elastic net (E-net), which linearly combined the penalty terms of the least absolute shrinkage and selection operator and ridge methods, was used to select features. This method minimized the residual sum of squares of the estimated errors plus the penalty term to select a model with the best trade-off between fit and complexity (23, 24). E-net was trained in the training set with tuning parameter α (0–1, step 0.1) and λ using 10-fold cross-validation, which followed the criterion of minimum standard deviation. Features with non-zero coefficients were finally selected from the model with optimal values of α and λ.

### Model Development: Kernel Support Vector Machine

A support vector machine (SVM) was used to develop the predictive 1p/19q co-deletion model. SVM is one of the most widely used machine learning algorithms. This classifier is based on Gaussian or Radial Basis Function kernel, which deals with non-linearity and higher dimensions and is aimed to find the best hyperplane that separates two groups of data points having a clear gap as wide as possible (25–29). The optimization attempts to minimize the loss of 10-fold cross-validation by varying the parameters, including box constraint and kernel scale parameter. The algorithms of E-net and kernel SVM were adopted from the MATLAB toolbox provided by the Statistics and Machine Learning Toolbox.

### Cross-Validation Strategy

The 1p/19q co-deletion status for WHO grade II gliomas was predicted using radiomic features while also considering age and gender as predictors. Nested cross-validation (CV) was considered as the gold standard method when an independent validation set was lacking. The nested CV makes full use of information without leaking and double dipping (30). To thoroughly assess the classifiers’ performance, a 10 × 10-fold nested CV scheme (**Figure 1**) was used in this study. Data were split into 10 sets; nine sets were used for training, whereas one non-overlapped set was used for testing, in each outer loop. Feature selection and model optimization of hyperparameter tuning were trained in each outer loop with an additional 10-fold CV, which was called the inner loop. After the feature selection and model optimization, we evaluated the model performance in the test set with the optimal model in each outer loop. This procedure was repeated 10 times and formed the outer loops of the nested CV. Finally, we built 10 different optimal models.

### Statistical Analysis

We used MATLAB 2019b (MathWorks, Natick, MA, USA) for data processing. The paired classification models, based on radiomic signatures, which underwent z-score transformation, were evaluated by receiver operating characteristic (ROC) analysis and precision-recall (P-R) analysis. We computed the area under the curve (AUC), accuracy, sensitivity [also known as true-positive rate (TPR) or recall)], specificity [also known as 1 − false-positive rate (1 − FPR)] from the ROC analysis, and precision, recall, and F1-score from the P-R analysis. The 95% confidence interval (CI) of performance was evaluated by the bootstrap method (1000 times sampling). We used point-biserial-correlation to compute the r and *p* values between the posterior probabilities of the 1p/19q co-deletion predicted by the SVM model and the true labels (31). To compare the posterior probability (transformed from the decision value of each model) of the kernel SVM model between the 1p/19q co-deletion and non-codeletion groups, a *t*-test was used, and a 1p/19q co-deletion was considered as 1 and non-codeletion as 0. A *p-*value < 0.05 was considered statistically significant.

## Results

### Clinical Characteristics

The clinical and pathological characteristics of all 157 patients are summarized in **Table 1**. Of the 157 enrolled patients with WHO grade II gliomas, 73 (46.5%) were women and the ages of patients ranged from 20 to 68 years (mean ± standard deviation, 41.6 ± 10.4 years). There were 76 (48.4%) patients with astrocytomas with mutant IDH, 16 (10.2%) patients with astrocytoma with wild-type IDH, and 65 (41.4%) patients with oligodendrogliomas with mutant IDH and 1p/19q codeletion. The mean ± standard deviation of tumor volume was 59.87 ± 52.74 cm^3^.

**Table 1:** Baseline demographics and clinical characteristics of patients.

Variable	Value
Number of Patients	157
Sex, %	
Male	84 (53.5%)
Female	73 (46.5%)
Age (years)*	41.6 ± 10.4
Pathology classification, %	
Diffuse astrocytoma, IDH-mutant	76 (48.4%)
Diffuse astrocytoma, IDH-wildtype	16 (10.2%)
Oligodendroglioma, IDH-mutant, and 1p/19q codeletion	65 (41.4%)
Tumor volume (cm^3^)*	59.87 ± 52.74

*Data are mean ± standard deviation.

IDH, isocitrate dehydrogenase; NOS, not otherwise specified.

### Radiomic Features Selection

A total of 431 radiomic features were extracted from each sequence, and a total of 1,293 radiomic features grouped by age and gender were screened by the E-net in the nested cross-validation. The number of selected signatures ranged from 11 to 103. Features that were selected in all of the 10 loops were considered to be the most valuable features. Six valuable radiomic features and age were selected in each outer loop (**Table 2**). Most of the radiomic features were textual (group 3) with wavelet transformed features (group 4) such as Informational Measure of Correlation_2, Long Run High Gray Level Emphasis_2, Long Run High Gray Level Emphasis_1, Short Run Low Gray Level Emphasis_1, Low Gray Level Run Emphasis_1, and Cluster Tendency. Only Skewness_1 extracted from CE-T1WI belonged to the wavelet transformation of first-order statistics (Group 1) features. We compared the value of age and z-scored value of six selected radiomic features between 1p/19q co-deletion and non-codeletion groups. The results showed that all the features except age (*p* = 0.2366) and CE-T1WI_Group 4_Cluster Tendency_6 (*p* = 0.7415) in the 1p/19q co-deletion group were significantly different (*p* < 0.05) from those in the 1p/19q non-codeletion groups.

**Table 2:** Selected valuable features.

Feature name	Selected times	p*
Age	10	0.2366
T2WI_Group 4_Informational Measure of Correlation_2	10	**0.0004**
T2WI_Group 3_Long Run High Gray Level Emphasis_2	10	**0.0319**
T2WI_Group 4_Long Run High Gray Level Emphasis_1	10	**<0.0001**
T1WI_Group 4_Short Run Low Gray Level Emphasis_1	10	**<0.0001**
T1WI_Group 4_Low Gray Level Run Emphasis_1	10	**<0.0001**
CE-T1WI_Group 4_Skewness_1	10	**<0.0001**
CE-T1WI_Group 4_Cluster Tendency_6	10	0.7415

*p-value of comparison between 1p/19q co-deletion and non-codeletion groups using unpaired t-test, the p-value < 0.05 were bolded.

### Model Performances

**Figure 2:**
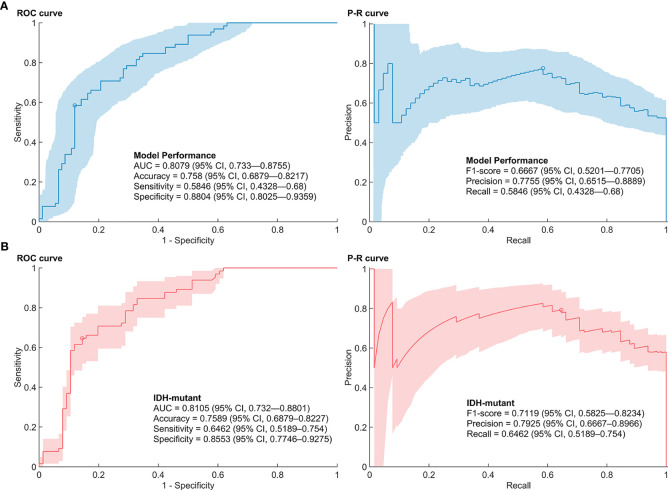
Performance of 1p/19q co-deletion predictive models. **(A)** Receiver operating characteristic (ROC) curve and precision-recall (P-R) curve of the predictive models in low-grade gliomas. **(B)** ROC curve and P-R curve of the predictive models in low-grade gliomas with mutant IDH.

The AUC of the SVM models with features selected by E-net in the nested CV was 0.8079 (95% CI, 0.733–0.8755) (**Figure 2**). The accuracy, sensitivity, specificity, precision, and F1-score of the prediction model were 0.758 (0.6879–0.8217), 0.5846 (0.4328–0.68), 0.8804 (0.8025–0.9359), 0.7755 (0.6515–0.8889), and 0.6667 (0.5201–0.7705), respectively. The range of box constraint and kernel scale parameters of SVM classifiers in the nested CV were 2.1544–1000 and 10–215.4435, respectively. The hyperparameters and performances of models in each outer loop are summarized in **Supplementary Table S1**. The misclassified number of patients in the patients with wild-type IDH was 4/16. We further performed the 1p/19q predictive models in patients with mutant IDH. The predictive models reached an AUC, accuracy, sensitivity/recall, specificity, precision and F1-score of 0.8105 (0.732—0.8801), 0.7589 (0.6879—0.8227), 0.6462 (0.5189—0.754), 0.8553 (0.7746—0.9275), 0.7925 (0.6667—0.8966) and 0.7119 (0.5825—0.8234).

**Figure 3:**
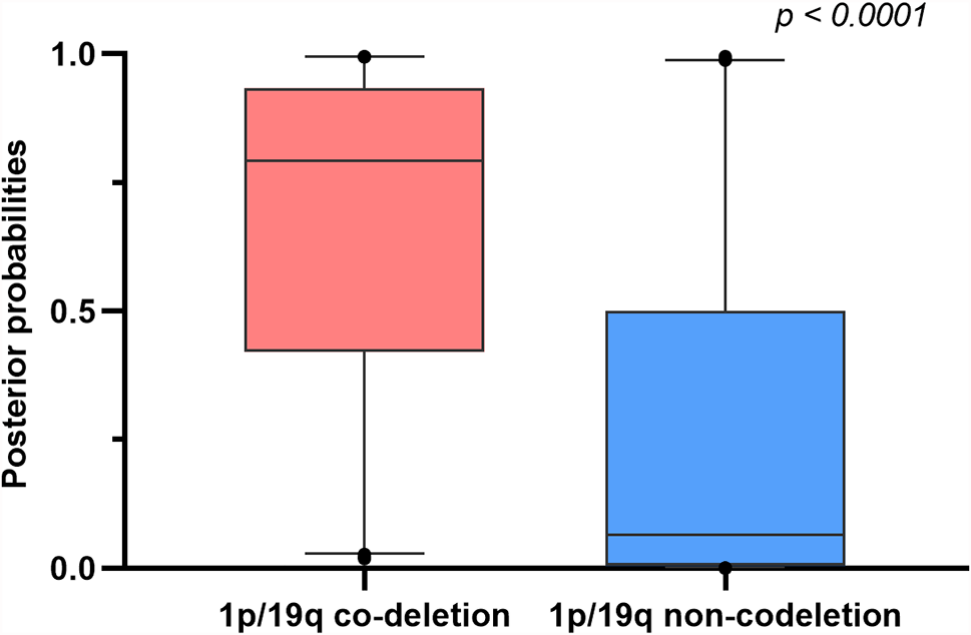
Boxplots comparing differences of posterior probabilities between 1p/19q co-deletion and non-codeletion groups.

The *p* and *r* values of the point-biserial-correlation were < 0.001 and 0.52, respectively. Moreover, the *p* value of *t* tests for comparison of posterior probabilities of groups, which was computed by the 1p/19q predictive model, for different 1p/19q status was < 0.001. The results indicated that these radiomic features could distinguish and predict the 1p/19q status of patients (**Figure 3**).

## Discussion

Patients with gliomas without 1p/19q co-deletions have poor prognostic survival outcomes (32, 33). Previous findings showed similar survival outcomes for patients with 1p/19q co-deletions who underwent subtotal resection. This information prevents damage to the eloquent cortices through total resection, which could potentially cause functional deficits (paralysis, aphasia, etc.) (8). Considering this, the prediction of 1p/19q co-deletion before surgery is useful in determining neurosurgery strategies. In this study, we built a machine learning model to preoperatively predict the status of 1p/19q co-deletion using radiomics analysis.

The predictive models of lower-grade gliomas (including WHO grade II and III gliomas) based on 1p/19q status and radiomics analysis can be clinically useful. Zhou et al. extracted textural features from preoperative MRIs of 165 patients of The Cancer Imaging Archive (TCIA) data set to develop a logistic regression model that achieves an AUC of 0.78 in predicting 1p/19q status of lower-grade gliomas (34). Further studies by this group showed a lower AUC of 0.72 of 1p/19q status prediction (random forest model) in all grade gliomas with an *IDH* mutation (35). Another study using nested LOOCV Xg-boost model exhibited a higher AUC (0.83 ± 0.03) in lower-grade gliomas (36), which may owe to the nested cross-validation. Considering information leakage of the validation group in the holdout method for CV and the small sample size, nested CV was considered the “gold standard” for building a predictive model (30). Previous studies developed a deep learning model including the features extracted from MRI, positron emission tomography, and computed tomography (CT), which showed an overall accuracy of 75.1% in the prediction of 1p/19q status in lower-grade gliomas (37). Since the predictive performance of these models was restrained by the small sample size, data augmentation was introduced to enlarge the size of the training set. Based on Cycle Generative Adversarial Network, multi-stream convolutional autoencoder and feature fusion are proposed for the prediction of 1p/19q co-deletion, which displayed an accuracy of 78.41% in low-grade gliomas (38). After adding 30-fold augmented data, another study improved the accuracy of the convolutional neural networks model from 78.3% to 87.7% (39). However, it ignored the global information of tumors since only three MRI slices were used.

Although the prediction of 1p/19q status can achieve good outcomes among patients with lower-grade gliomas (40), building an effective predictive model for low-grade gliomas, which only contain WHO grade II gliomas, is complicated primarily because of the limited sample size. However, the radiomic features of low-grade gliomas are different from those in WHO grade III in conventional MRI sequences (41, 42). To avoid bias caused by the differences of radiomic features in different WHO grades, our study developed a radiomic-based SVM model to predict 1p/19q co-deletions in WHO grade II gliomas. Although the sample size was limited, it allowed the results to be more consistent. Our model showed a similar performance to the machine-learning and deep-learning models mentioned above, with an overall AUC of 0.8079 and an accuracy of 0.758 (34, 35, 38, 39). We specifically analyzed the predictive model in the subgroup analysis of gliomas with mutant IDH, which excluded the influence of gliomas with wild-type IDH, and found a similar performance value. We further compared the predictive probabilities for patients with 1p/19q co-deletion and non-codeletion, and the result exhibited a significant difference. These results indicated that radiomics analysis combined with machine learning can potentially predict 1p/19q mutation in WHO grade II gliomas.

We extracted six valuable radiomic signatures from each outer loop for our predictive model. Gliomas with 1p/19q co-deletion showed a lower homogeneity than those without (43). Our findings confirmed that a lower Informational Measure of correlation in T2WI is exhibited in patients with 1p/19q co-deletion, which shows a positive correlation with homogeneity degrees (44). Moreover, cluster tendency is another feature used to reveal the degree of homogeneity, which represents the measure of the groupings of voxels with similar gray-level values. Our results showed that patients with 1p/19q co-deletion had a lower Cluster Tendency than those patients without. This finding indicated that the degree of homogeneity in an oligodendroglioma is lower compared with an astrocytoma (45). Besides, features belonging to Gray Level Run Length are often applied to distinguish malignant and benign brain tumors (46). In our model, these features (Long Run High Gray Level Emphasis and Short Run Low Gray Level) were crucial for predicting the status of 1p/19q co-deletion due to the difference in prognostic outcomes between oligodendroglioma and astrocytoma (1, 3). Furthermore, skewness, which was significantly different in patients with or without 1p/19q co-deletion, was a classical feature used in distinguishing brain tumors and in the differentiation of glioblastomas and primary central neuro-lymphoma (8, 47, 48).

There are several limitations to the present study. First, our model was generated using retrospectively collected data. Although we performed nested CV to minimize the potential bias, the lack of an external validation data set limited the generalizability of our models. In addition, since our small sample size limited the efficacy of our model, we plan to develop a model based on a larger population combined with independent external validation. Furthermore, we would like to develop a multi-model radiological data-based classifiers in the future, which would include T2-FLAIR, diffusion-weighted imaging, and CT (49–53).

In conclusion, we developed a nested cross-validation machine learning model with efficacy and robust performance, which displayed an AUC of 0.8079 and an accuracy of 0.758. Our results revealed the potential clinical utility of radiomics analysis in the preoperative prediction of 1p/19q status, which can aid in preoperative genomic marker prediction and making customized neurosurgery plans and glioma management before postoperative pathology.

## Data Availability Statement 

The raw data supporting the conclusions of this article will be made available by the authors, without undue reservation.

## Ethics Statement

The studies involving human participants were reviewed and approved by IRB of Beijing Tiantan Hospital. The patients/participants provided their written informed consent to participate in this study.

## Author Contributions

Study conception and design: YW and LW. Acquisition of data: XL, YCL, YKL, CZ, QZ, TL, and HZ. Analysis and interpretation of data: ZS, ZF, SF, YL, and YW. Drafting of manuscript: ZF and ZS, Critical revision: YW and LW. All authors contributed to the article and approved the submitted version.

## Funding

This study was supported by grants from the National Natural Science Foundation of China (grant 82072786), the Beijing Municipal Natural Science Foundation (7202021), and Capital’s Funds for Health Improvement and Research (CFH 2018-2-1072).

## Conflict of Interest

The authors declare that the research was conducted in the absence of any commercial or financial relationships that could be construed as a potential conflict of interest.

The handling editor HB declared a past co-authorship with one of the authors YW.
