# Supplementary material for: Preoperative Radiomics Analysis of 1p/19q Status in WHO Grade II Gliomas
Source: Front Oncol. 2021 Jul 6;11:616740. doi: 10.3389/fonc.2021.616740 (PMC8290517; doi:10.3389/fonc.2021.616740)
Supplement: Supplementary file 2 [file Table_1.docx]

**Supplementary Table S1**. hyperparameters and performances of SVM predictive models in each outer loop.

| Loop | Box constraint | Kernel scale | AUC | Accuracy | Sensitivity/ recall | Specificity | Precision | F1-score |
| --- | --- | --- | --- | --- | --- | --- | --- | --- |
| 1 | 2.15 | 10 | 0.8334  (0.5461, 1) | 0.8667  (0.6667, 1) | 0.8334  (0.5, 1) | 0.8889  (1, 0.6364) | 0.8334  (0.5, 1) | 0.8334  (0.5, 1) |
| 2 | 46.42 | 10 | 0.85  (0.6, 1) | 0.875  (0.6875, 1) | 1  (1, 1) | 0.8  (1, 0.5556) | 0.75  (0.4286, 1) | 0.8572  (0.6, 1) |
| 3 | 2.15 | 10 | 0.9167  (0.69, 1) | 0.9375  (0.8125, 1) | 0.8334  (0.5, 1) | 1  (1, 1) | 1  (1, 1) | 0.9091  (0.6667, 1) |
| 4 | 46.42 | 46.42 | 0.9683  (0.8594, 1) | 0.9375  (0.8125, 1) | 0.8572  (0.5, 1) | 1  (1, 1) | 1  (1, 1) | 0.9231  (0.6667, 1) |
| 5 | 46.42 | 46.42 | 0.9366  (0.7643, 1) | 0.9375  (0.8125, 1) | 0.8572  (0.5, 1) | 1  (1, 1) | 1  (1, 1) | 0.9231  (0.6667, 1) |
| 6 | 1000 | 215.44 | 0.8096  (0.5528, 1) | 0.8125  (0.625, 1) | 1  (1, 1) | 0.6667  (1, 0.3334) | 0.7  (0.4, 1) | 0.8236  (0.5715, 1) |
| 7 | 10 | 46.42 | 0.8889  (0.625, 1) | 0.875  (0.6875, 1) | 0.8572  (0.5, 1) | 0.8889  (1, 0.6516) | 0.8572  (0.5585, 1) | 0.8572  (0.5277, 1) |
| 8 | 46.42 | 46.42 | 0.635  (0.3175, 0.9366) | 0.75  (0.5625, 0.9375) | 1  (1, 1) | 0.5556  (0.875, 0.2) | 0.6364  (0.3637, 0.9046) | 0.7778  (0.5334, 0.9499) |
| 9 | 2.15 | 10 | 0.5  (0.1955, 0.8393) | 0.6  (0.3334, 0.8) | 0  (0, 0) | 1  (1, 1) | 0  (0, 0) | 0  (0, 0) |
| 10 | 46.42 | 46.42 | 0.963  (0.84, 1) | 0.9334  (0.8, 1) | 1  (1, 1) | 0.8889  (1, 0.6307) | 0.8572  (0.5, 1) | 0.9231  (0.6667, 1) |

* The 95% CI of model performance was measured by bootstrap.
